# Serum 25-hydroxyvitamin D and bone turnover markers in Palestinian postmenopausal osteoporosis and normal women

**DOI:** 10.1007/s11657-017-0306-7

**Published:** 2017-01-26

**Authors:** Akram Kharroubi, Elias Saba, Riham Smoom, Khaldoun Bader, Hisham Darwish

**Affiliations:** 1 Faculty of Health Professions, Al-Quds University, Jerusalem, Palestine; 2 Palestinian Osteoporosis Prevention Society [POPS], Bethlehem, Palestine; 3 Medical Research Center, Al-Quds University, Jerusalem, Palestine; 4 Faculty of Public Health, Al-Quds University, Jerusalem, Palestine; 5 Faculty of Allied Medical Sciences, Arab American University—Jenin AAUJ, Jenin, Palestine

**Keywords:** Postmenopausal osteoporosis, Bone markers, 25-Hydroxyvitamin D, Palestinian women, Bone turnover

## Abstract

***Summary*:**

This study evaluated the association of vitamin D and bone markers with the development osteoporosis in Palestinian postmenopausal women. Even though vitamin D deficiency was very high for the recruited subjects, it was not associated with osteoporosis except for bones of the hip. Age and obesity were the strongest determining factors of the disease.

**Purpose:**

The purpose of this study was to investigate the association of bone mineral density (BMD) with serum vitamin D levels, parathyroid hormone (PTH), calcium, obesity, and bone turnover markers in Palestinian postmenopausal women.

**Methods:**

Three hundred eighty-two postmenopausal women (≥45 years) were recruited from various women clinics for BMD assessment (131 women had osteoporosis and 251 were normal and served as controls). Blood samples were obtained for serum calcium, PTH, 25(OH)D, bone formation (N-terminal propeptide (PINP)), and bone resorption (serum C-terminal telopeptide of type I collagen (CTX1)) markers.

**Results:**

Women with osteoporosis had statistically significant lower mean weight, height, body mass index (BMI), and serum calcium (*p* < 0.05) compared to controls. No significant differences were detected between the mean values of bone turnover markers (CTX and PINP), 25(OH)D, and PTH of the two groups. Women with vitamin D deficiency (severe and insufficiency) represented 85.9% of the study subjects. Multiple and logistic regression showed that age and BMI significantly affected BMD and vitamin D had a significant association with BMD only at the lumbar spine. BMI was positively correlated with BMD and PTH but negatively correlated with vitamin D. Logistic regression showed that the odds ratio (OR) for having osteoporosis decreased with increasing BMI (overweight OR = 0.11, *p* = 0.053; obese OR = 0.05, *p* = 0.007).

**Conclusions:**

There was no direct correlation between BMD and PTH, bone turnover markers, and vitamin D except at the lumbar spine. A negative correlation between BMD and age and a positive correlation with BMI were observed. The protective effect of obesity on osteoporosis was complicated by the effect of obesity on vitamin D and PTH.

## Introduction

Osteoporosis is a widespread disease characterized by significant decrease in bone quality as a consequence of deterioration in bone microarchitecture and low bone mass [1, 2]. Primary osteoporosis is common in postmenopausal women but can also affect men at an older age. Common bone fractures constitute the major cause of high mortality and morbidity among patients with mounting cost on health services [3]. Bone mineral density (BMD) is determined by several non-genomic (nutritional, hormonal) and genetic factors [4, 5]. Genome wide association studies provided positive association between certain loci in the genome and osteoporosis [6].

Vitamin D plays a central role in bone formation and remodeling [7–9]. Several studies have documented the importance of maintaining adequate levels of serum vitamin D to protect against bone fracture [10–15]. Vitamin D deficiency is currently a major health concern worldwide [7, 16, 17]. Although the Middle East countries enjoy sunshine year around, their populations suffer from significant vitamin D deficiency in all ages and have one of the highest rates of rickets in the world [10, 15, 18, 19]. Several factors contribute to the development of vitamin D deficiency and its negative physiological impact on bones including decreased dietary intake, inadequate production of the vitamin in the skin, and disturbances in the production of the active hormonal form of vitamin D [10, 15, 20–23]. Recently, vitamin D deficiency, which is common among the Saudi Arabian population, reached 41–64% among young females 12–18 years old [24–27]. In addition to the vitamin D status, mutations in the vitamin D receptor markedly contribute to the complications of vitamin D deficiency on bone health [28, 29].

Bone remodeling is a slow and life-long active process influenced by vitamin D receptor activity in bone cells [30]. The magnitude of bone remodeling is evaluated through the measurement of specific bone turnover markers including serum carboxy-terminal telopeptide (s-CTX) and the N-terminal propeptide (PINP) of procollagen type I [31–33].

Previously, we reported relatively high prevalence of postmenopausal osteoporosis in the Palestinian population that was associated with poor knowledge and awareness about the various risk factors of the disease [34]. Body mass index positively correlates with BMD [35], while very low BMI represents an independent osteoporotic fracture risk [36]. However, obesity does not protect against all osteoporotic fractures [37] and bone recovery after fractures is worse among obese compared to non-obese individuals [38].

In the present study, we investigated the correlation between BMD at various specific skeletal sites (total hip, lumbar L1–L4 spine, and femoral neck) with serum levels of 25(OH)D, parathyroid hormone (PTH), calcium, specific markers of bone formation, and bone resorption, in addition to other non-genomic factors including age, height, weight, and BMI among Palestinian postmenopausal women. The effect of obesity on osteoporosis, as reflected by the relationship between obesity and BMD, was also analyzed.

## Methods

### Study subjects

In this cross-sectional study, the subjects comprised of 382 postmenopausal women (ages ≥45 years) were recruited from various clinics and community centers from the central part of the West Bank region of Palestine. All recruited subjects were not previously diagnosed with bone problems or suffer from bone-related health conditions and had no previous BMD assessment. In addition, none of the subjects suffered from any medical complication that affected bone health status and were not using any prescription drugs or food supplements (including vitamin D and calcium) that affected their general bone status. All subjects were referred to a special clinic and were medically checked for their vital signs, their height and weight were recorded, and BMI was calculated. All subjects were interviewed by the medical staff of the clinic to fill a special questionnaire concerning their life style and general health information that was designed, tested, and used previously by our group [34]. Blood samples were collected from all subjects; serum was immediately separated and stored frozen until the level of specific serum bone-related markers was measured. All study subjects were required to sign a consent form declaring their agreement to participate in the study. Ethical approval for the study protocol was obtained from the Research Ethics Committee of Al-Quds University in Palestine.

### Bone mineral density and serum bone markers assessment

BMD was assessed using dual energy x-ray absorptiometry DXA (Lunar Prodigy GE) at the total hip, femoral neck, and lumbar (L1–L4) spine. Measurements of BMD were very precise (coefficient of variation (CV) = 0.28%). Serum 25(OH)D (CV = 2.33% and 6.45% for intra- and inter-assay, respectively) and PTH (CV = 2.08% and 1.75 for intra- and inter-assay, respectively) were measured by chemiluminescence using Architect 1000 (Abott, USA). Serum calcium was measured by a calorimetric end point method. CTX and PINP (CV < 10%) were measured by ELISA. Osteoporosis is defined as having a *T* score ≤ −2.5 at any of the three tested sites.

### Statistical analysis

SPSS program was utilized for all data entry and related analysis that included frequencies, descriptive statistics, and bivariate correlations (Pearson’s). Simple linear, multiple, and logistic regression analyses were also performed on the data to assess the correlation between BMD and the various indicated variables. Chi-square test (two-sided) was performed in order to identify differences in categorical variables between subgroups and independent *t* test for continued variable. One-way analysis of variance (ANOVA) was undertaken to assess differences in means of calcium and PTH by vitamin D groups. Significance levels of less than 5% were considered significant.

## Results

Table 1 of univariate analysis showed that mean values of weight, height, and BMI were significantly lower (*p* < 0.0001) in women with osteoporosis, while their mean age was significantly higher compared to normal control subjects (68.2 vs. 61.3 years, respectively, *p* < 0.0001). The mean values of BMI for both groups were above 30 kg/m^2^, which indicated that osteoporosis and control subjects were obese. No significant differences were detected between mean values of calcium, 25(OH)D, PTH, and bone turnover markers (CTX1 and PINP) in women with osteoporosis compared to controls. Due to the fact that there was a significant difference in the mean age between control and osteoporosis groups, the differences between the indicated markers (vitamin D, Ca, PTH, CTX1, and PINP) were reevaluated after taking a subset of the control group with a mean age that matches the mean age of the osteoporosis group. Similar results were obtained that ruled out the differences between the indicated markers being due to age (data not shown).

**Table 1:** Mean values related to osteoporosis in normal and osteoporosis women

Parameter	Control	Osteoporosis	*p* value
	Mean ± STD (*N*)	Mean ± STD (*N*)	
Age	61.3 ± 7.98 (251)	68.2 ± 8.55 (131)	<0.0001
Weight Kg	82.2 ± 13.39 (251)	71.4 ± 11.82 (131)	<0.0001
Height cm	157.2 ± 7.28 (251)	153.6 ± 7.51 (131)	<0.0001
BMI	33.1 ± 5.28 (247)	30.4 ± 5.29 (131)	<0.0001
CTX1 pg/ml	6526 ± 4323 (151)	6857 ± 4313 (121)	0.531
PINP μg/l	549 ± 330 (150)	567 ± 342 (120)	0.654
Ca mg/dl	9.38 ± .50 (157)	9.28 ± 0.56 (122)	0.121
Vitamin D ng/ml	14.1 ± 4.85 (154)	13.6 ± 4.82 (123)	0.396
PTH pg/ml	70.7 ± 32.3 (155)	75.2 ± 33.3 (121)	0.253
Total hip BMD gm/cm^2^	0.979 ± 0.131 (242)	0.780 ± 0.114 (128)	<0.0001
Femoral neck BMD gm/cm^2^	0.888 ± 0.125 (248)	0.695 ± 0.091 (130)	<0.0001
Lumbar spine BMD gm/cm^2^	1.100 ± .154 (248)	0.858 ± 0.139 (130)	<0.0001
Total hip *T* score	−0.220 ± 1.030 (242)	−1.817 ± 0.911 (128)	<0.0001
Femoral neck *T* score	−1.122 ± 0.829 (244)	−2.466 ± 0.648 (130)	<0.0001
Lumbar spine *T* score	0.683 ± 1.262 (247)	−2.708 ± 1.179 (130)	<0.0001

*p* values are independent *t* test

*STD* standard deviation, *N* number of subjects, *BMI* body mass index, *PINP* procollagen type I N propeptide, *CTX1* serum C-terminal telopeptide of type I collagen, *PTH* parathyroid hormone, *BMD* bone mineral density

Table 2 showed that 14.1% of the study subjects had severe vitamin D deficiency (< 10 ng/ml 25(OH)D with mean ± SD 8.51 ± 1.28 ng/ml), whereas 71.8% had vitamin D insufficiency or borderline [25(OH)D 10 to < 20 ng/ml with mean ± SD 13.26 ± 2.20 ng/ml]. Subjects with normal (vitamin D sufficient) represented 14.1% [25(OH)D ≥ 20 ng/ml with mean ± SD 24.55 ± 3.60 ng/ml] [7, 39]. Chi-square analysis showed no statistically significant difference in the prevalence of vitamin D groups between osteoporosis and control subjects. Table 2 also showed the levels of calcium and PTH in subjects in the three vitamin D groups. ANOVA showed that mean serum calcium levels were significantly higher in subjects with vitamin D sufficiency compared to serum calcium levels in subjects with severe vitamin D deficiency or vitamin D insufficiency (*p* = 0.022). However, it should be indicated that serum calcium levels in the three groups were within the normal range. When PTH levels were compared in the three groups, ANOVA showed that mean values of PTH levels were significantly lower in subjects with vitamin D sufficiency (*p* < 0.0001) compared to vitamin D insufficiency or severe vitamin D deficiency subjects. The mean values of PTH were not statistically different between severe vitamin D deficiency and vitamin D insufficiency subjects. Approximately, 27% of recruited subject had previous accident that caused broken bones with no difference between osteoporotic and control subjects.

**Table 2:** Frequency of subjects and serum calcium and parathyroid hormone levels in different vitamin D groups

Parameter	25(OH)D_3_			*p* value
	≥20 ng/ml	10 to <20 ng/ml	<10 ng/ml	
	Normal (sufficiency)	Insufficiency (borderline)	Severe deficiency	
Osteoporosis *N* (%)	15 (12%)	94 (75%)	16 (13%)	0.52
Control *N* (%)	25 (16%)	110 (69%)	24 (15%)	
Total *N* (%)	40 (14.1%)	204 (71.8%)	40 (14.1%)	
Ca mg/dl	9.55 ± 0.52 (39)	9.30 ± 0.51 (202)	9.21 ± 0.59 (38)	0.022
PTH pg/ml	49.3 ± 19.8 (39)	75.2 ± 31.8 (198)	83.3 ± 37.6 (39)	<0.0001

PTH and Ca values are mean ± STD (*N*)

*N* number of subjects, *PTH* parathyroid hormone, *Ca* calcium

**Fig. 1:**
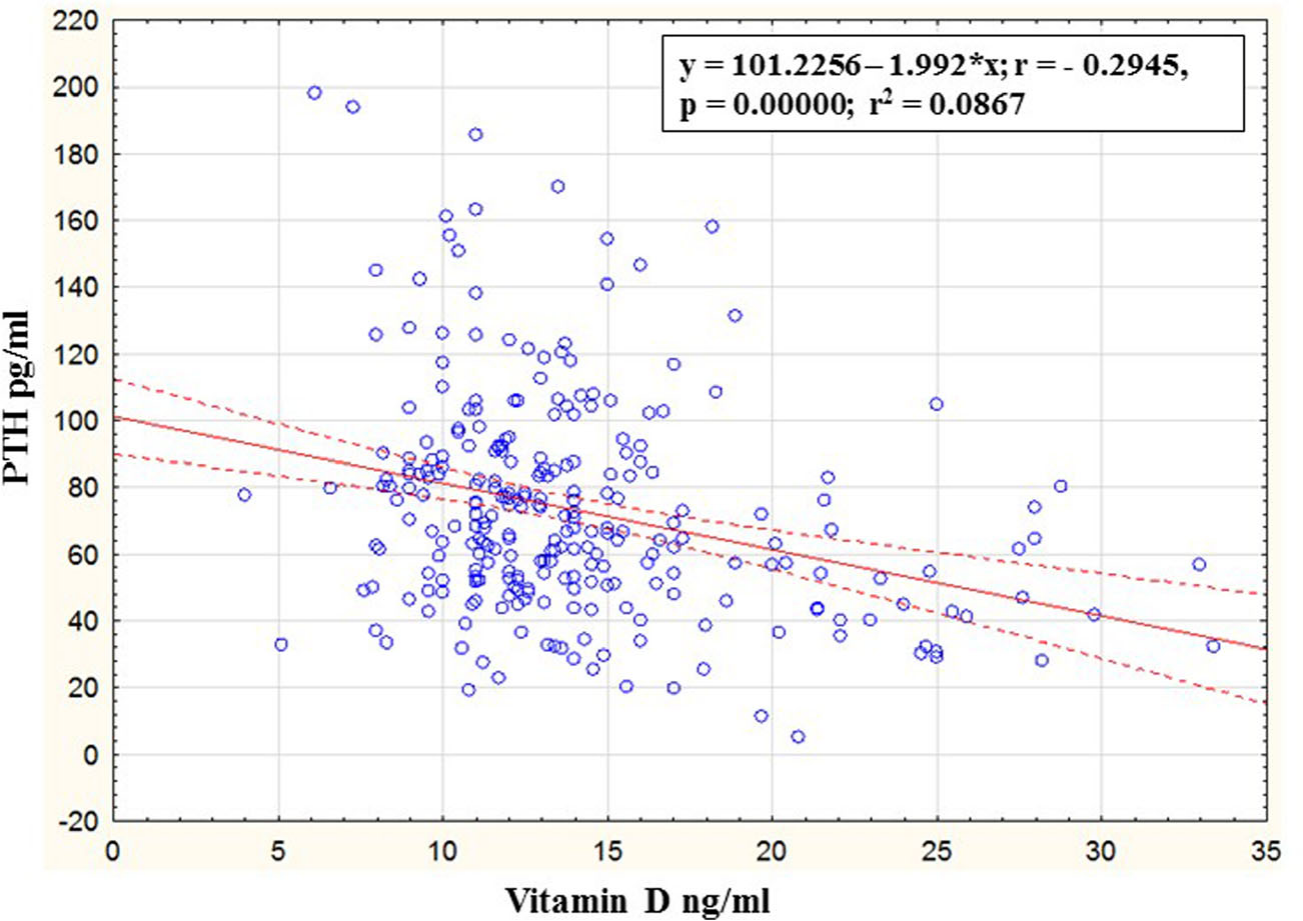
Scatter plot of parathyroid hormone against vitamin D

A scatter plot showing the relationship between vitamin D and PTH is depicted in Fig. 1. Vitamin D was negatively correlated with PTH in all subjects (*r* = −0.295, *p* < 0.01) as well as in osteoporosis subjects (*r* = −0.30, *p* < 0.01).

Table 3 showed the Pearson correlation coefficients of the parameters measured in the tested subjects. Serum 25(OH)D was significantly negatively correlated with PTH, weight, and BMI and positively correlated with calcium. No statistically significant correlations were detected between vitamin D and BMD at the three indicated sites (total hip, femoral neck, and lumbar L1–L4 spine), as well as with age, height, bone resorption (CTX1), and bone formation (PINP) markers. Age was negatively correlated with BMD (total hip, femoral neck, and lumbar L1–L4 spine), height, weight, and BMI. Pearson correlation showed the markers for bone resorption (CTX1) and bone formation (PINP) were positively correlated (*r* = 0.529, *p* < 0.01). This correlation between bone-remodeling markers was very strong in subjects with osteoporosis (*r* = 0.887, *p* < 0.000) whereas no correlation was seen in normal control subjects (*r* = 0.03, *p* = 0.74). Among control subjects, PINP was negatively correlated with femoral neck BMD (*r* = −0.199, *p* = 0.015) and height (*r* = −0.173, *p* = 0.034) and positively correlated with BMI (*r* = 0.190, *p* = 0.021). Calcium levels were positively correlated with vitamin D levels and BMD of total hip, whereas calcium was negatively correlated with PTH. These correlations, despite being significant, were not strong. Body mass index was positively correlated with BMD (in total hip, femoral neck, and lumbar L1–L4 spine) and PTH and negatively correlated with vitamin D.

**Table 3:** Pearson correlation between different bone-remodeling parameters related to osteoporosis

Parameter	Age years	Weight Kg	Height cm	BMI Kg/m^2^	PINP μg/l	CTX1 pg/ml	Ca mg/dl	Vit. D ng/ml	PTH pg/ml	BMD hip	BMD neck	BMD L1–L4
Age years	1											
Weight Kg	−0.263**	1										
Height cm	−0.219**	0.223**	1									
BMI Kg/m^2^	−0.134**	0.841**	−0.305**	1								
PINP μg/l	0.066	−0.005	−0.076	0.056	1							
CTX1 pg/ml	0.038	−0.007	−0.048	−0.024	0.529**	1						
Ca mg/dl	−0.083	0.008	0.028	0.007	−0.014	−0.106	1					
Vit. D ng/ml	−0.039	−0.148*	0.035	−0.148*	0.071	0.008	0.169**	1				
PTH pg/ml	−0.014	0.104	−0.109	0.129*	0.078	0.112	−0.132*	−0.295**	1			
BMD hip gm/cm^2^	−0.431**	0.444**	0.192**	0.321**	−0.059	−0.059	0.122*	0.001	−0.024	1		
BMD neck gm/cm^2^	−0.459**	0.394**	0.233**	0.255**	−0.096	−0.004	0.072	0.014	−0.011	0.866**	1	
BMD L1–L4 gm/cm^2^	−0.276**	0.425**	0.230**	0.279**	−0.073	−0.102	0.111	0.078	−0.045	0.649**	0.625**	1

*Correlation is significant at the 0.05 level (two-tailed)

**Correlation is significant at the 0.01 level (two-tailed)

*BMD hip* total hip BMD, *BMD neck* femoral neck BMD, *BMD L1–L4* lumbar (L1–L4) spine BMD, *BMD* bone mineral density, *BMI* body mass index, *PINP* procollagen type I N propeptide, *CTX1* serum C-terminal telopeptide of type I collagen, *PTH* parathyroid hormone

Correlations within the osteoporosis group were also investigated. No significant correlations were seen between calcium and vitamin D, calcium and PTH, BMI and vitamin D, BMI and PTH, BMI and femoral neck BMD, BMI and age, and height and BMD at the three sites. Lumbar spine BMD was positively correlated with vitamin D (*r* = 0.193, *p* = 0.033), negatively correlated with PTH (*r* = −0.198, *p* = 0.030), and not correlated with height and age.

To determine which variables could predict the BMD level when controlling for the effect of others at each of the tested locations, multiple regression was performed on all variables (Table 4). The results showed that irrespective of the location, both age and weight of the participants appeared to be significant determinants of BMD. Weight appeared to have a positive medium effect on BMD at all locations, whereas age was a negative predictor that lost some of its correlation strength with BMD in the lumbar spine. All other variables did not show any association with BMD at any tested location, except for vitamin D, which appeared to have an effect on the lumbar spine. The models in Table 4 suggested that the variables entered in the analysis had the strongest predictive effect on total hip BMD (*R* ^2^ = 0.34) but less predictive effect on BMD of femoral neck (*R* ^2^ = 0.29) or lumbar spine (*R* ^2^ = 0.225).

**Table 4:** Association of bone marker variables with BMD by location using multiple regression analysis

BMD gm/cm^2^	Model *R* ^2^		CTX1 pg/ml	PINP μg/l	Ca mg/dl	Vit. D ng/ml	PTH pg/ml	Age years	Weight Kg	Height cm
Total hip	0.340	*p* value	0.85	0.510	0.969	0.946	0.124	*<0.0001*	*<0.0001*	0.505
		Adjusted *R*	−0.013	0.048	0.003	−0.005	−0.112	*−0.34*	*0.425*	−0.049
Femoral neck	0.295	*p* value	0.096	0.306	0.508	0.348	0.690	*<0.0001*	*<0.0001*	0.711
		Adjusted *R*	0.119	−0.073	−0.047	0.067	−0.029	*−0.364*	*0.346*	0.027
Lumbar spine	0.225	*p* value	0.136	0.778	0.808	*0.038*	0.217	*0.021*	*<0.0001*	0.491
		Adjusted *R*	−0.107	0.02	0.018	*0.149*	−0.089	*−0.166*	*0.335*	0.05

*BMD* bone mineral density, *PINP* procollagen type I N propeptide, *CTX1* serum C-terminal telopeptide of type I collagen, *PTH* parathyroid hormone, *Ca* calcium, *Vit. D* 25(OH)D_3_

To test which variable has a significant effect that could be a determinant on the probability that the tested subject would become osteoporotic, binary logistic regression was carried out (Table 5). The results showed that age and obesity status of the subject could be important determinants of osteoporosis. Age progression appeared as a risk factor with odds ratio (OR) = 1.1 (1.05–1.15). Obesity status was protective against osteoporosis development with a borderline effect for being overweight (OR = 0.11, *p* = 0.053) and a very clear effect for being obese (OR = 0.05, *p* = 0.007). The protective effect of obesity on the development of osteoporosis was only seen in the total hip of obese subjects (OR = 0.195, *p* = 0.04) but not in overweight subjects. Among all models tested, the overall osteoporotic status model was the strongest in terms of its predictive power and joint effect of the significant variables on the change in the status compared to individual sites. Age was the only significant predictor at all sights and the only significant predictor for osteoporotic development in the femoral neck and lumbar spine.

**Table 5:** Logistic regression analysis for predictors of osteoporosis occurrence by location

	Osteoporosis occurrence at any location		Total hip		Femoral neck		Lumbar spine	
*R* ^2^ of the model	0.287		0.162		0.236		0.25	
	*p* value	OR (95% CI)	*p* value	OR (95% CI)	*p* value	OR (95% CI)	*p* value	OR (95% CI)
CTX1 pg/ml	0.280	1.00	0.52	1.00	0.41	1.00	0.07	1.00
PINP ug/L	0.437	1.00	0.23	1.00	0.29	1.00	0.48	1.00
Ca mg/dl	0.878	0.94 (0.46–1.91)	0.54	0.811 (0.41–1.60)	0.25	1.66 (0.69–3.98)	0.88	0.943 (0.41–2.14)
PTH pg/ml	0.078	1.00	0.26	1.00	0.26	1.00	0.99	1.00
Age	*˂0.001*	*1.10* (*1.05–1.15*)	*0.005*	*1.058* (*1.01–1.10*)	*0.002*	*1.09* (*1.03–1.15*)	*˂0.001*	*1.10* (*1.05–1.16*)
Vit. D status (trend)	(0.307)		(0.46)		(0.60)		(0.20)	
Insufficient	0.370	1.58 (0.57–4.36)	0.80	1.131 (0.43–2.95)	0.32	0.48 (0.11–2.02)	0.07	2.62 (0.90–7.61)
Deficient	0.835	0.87 (0.25–3.04)	0.51	0.672 (0.20–2.20)	0.48	0.54 (0.09–2.99)	0.19	2.43 (0.63–9.37)
Obesity status (trend)	(*0.004*)		(*0.01*)		(0.006)		(0.41)	
Overweight	0.053	0.11 (0.01–1.03)	0.34	0.452 (0.08–2.33)	0.95	1.07 (0.10–11.39)	0.99	1.00
Obese	*0.007*	*0.05* (*0.006–0.45*)	*0.04*	*0.195* (*0.04–0.95*)	0.14	0.20 (0.02–1.74)	0.99	1.00

*PINP* procollagen type I N propeptide, *CTX1* serum C-terminal telopeptide of type I collagen, *PTH* parathyroid hormone, *Ca* calcium, *Vit. D* 25(OH)D_3_, *OR* odds ratio

## Discussion

The present data showed vitamin D deficiency to be common among Palestinian postmenopausal women which is consistent with the widespread vitamin D deficiency in Palestine [39] and Middle Eastern Arab countries [15, 27, 40]. The level of vitamin D depends on several factors including diet, exposure to sun, age, feeding habits, life style, metabolic, genetic, and other environmental factors [7, 41]. In Palestine, like the rest of Middle Eastern countries, adequate sunshine is available throughout the whole year; however, exposure of women to sunlight is limited due to cultural and social factors. In addition, poor awareness of the disease, absence of screening programs, and poor advice on the need for regular consumption of dairy products and vitamin D-rich food largely contribute to the observed high vitamin D deficiency among postmenopausal women. Moreover, poor socioeconomic conditions including limited income and large family size add additional complications to the problem [34]. In addition, iron deficiency is common among the Palestinian population [39] and intestinal absorption of vitamin D is influenced by iron metabolism which provides an additional factor in determining serum vitamin D levels [42].

Clearly, the inverse correlation between vitamin D level and PTH was evident. Our results showed no correlation between vitamin D and BMD levels and mean vitamin D levels were not significantly different between osteoporotic and control groups. However, a positive correlation between vitamin D and lumbar spine BMD in Pearson correlation of osteoporosis postmenopausal women subgroup and in multiple regression analysis was seen and indicated differential effect of vitamin D on BMD at this site. Even though multiple regression analysis showed significant effect on BMD of lumbar spine, logistic regression analysis showed this effect was not enough to affect the status of osteoporosis among tested subjects. This is in agreement with previous studies in Lebanon, Saudi Arabia, United Arab Emirates, and Iranian postmenopausal women that found no correlation between 25(OH)vitamin D and BMD [10, 40, 43–45] and in other counties [12, 46–48]. The lack of correlation between BMD and serum 25(OH)vitamin D does not exclude a correlation of BMD with intracellular active 1,25(OH)_2_ D_3_ produced in all tissues including bone cells that exerts its function through an autocrine effect [49, 50].

The cutoff value for vitamin D using PTH levels as reference was 14.3 ng/ml which represents the optimum vitamin D levels that prevents secondary hyperparathyroidism among the tested subjects. This value is comparable to the vitamin D cutoff value (15.4 ng/ml) determined by similar analysis in a study of hypovitaminosis among college students in a country in the same region [51] and in contrast to 30 ng/ml in the Spanish community [52]. These reflection points are not reliable and are not used to derive desirable vitamin D levels. The increase in PTH due to a decrease in vitamin D that was not reflected dramatically on calcium levels that remained within the normal range is probably due to the effect PTH on bone and kidney and the effect of vitamin D on calcium absorption. As expected, the observed effect of vitamin D on PTH in this study is mediated through calcium since calcium levels are significantly lower in subjects with severe vitamin D deficiency leading to increased PTH levels.

The negative correlation of age with BMD was obvious at the three bone sites (total hip, femoral neck, and lumbar L1–L4 spine). This is consistent with the fact that during the process of bone remodeling, bone formation takes less time than resorption [2, 53, 54]. Similar to the effect of vitamin D, no correlation was evident between BMD and serum calcium, PTH, PINP (bone formation marker), and CTX1 (bone resorption marker). However, a significantly higher level of bone remodeling was evident in the osteoporosis group as reflected by the high correlation between PINP and CTX1 in these subjects compared to the absence of correlation in the control group between the indicated markers. These results are consistent with the understanding that BMD is determined by complex interactions between genetic and non-genetic factors that play a role in determining the final bone health status [2]. Eventually, higher rate of bone remodeling in osteoporosis may lead to reduced stability in bone strength and could provide an explanation for the higher fracture risk in osteoporotic compared to non-osteoporotic individuals [13].

An additional complication in our understanding of osteoporosis was the effect of obesity on bone health. Our data showed that obesity had a positive correlation with BMD in the spine, total hip, and femoral neck and was protective against osteoporosis which is consistent with several previous reports [34, 55–60]. On the other hand, our data also showed that obesity had a negative correlation with vitamin D and a positive correlation with PTH which may indicate that obesity is a risk factor for osteoporosis which has been also shown by other investigators [61–65]. The overall effect of obesity seems to be protective since the number of osteoporotic subjects among obese postmenopausal women was significantly lower than that in overweight and normal weight subjects and the odds ratio of being osteoporotic is lower in obese compared to overweight and normal weight. Other investigators found that obesity was correlated with increased selective bone fracture risk independent of the positive effect on BMD levels which indicate that bone health and osteoporosis development result from a complicated multifactorial process [63, 66–68]. Furthermore, since obesity constitutes a strong risk factor for diabetes, the associated low vitamin D levels, decreased insulin secretion, and or decreased insulin sensitivity contribute to the complexity of the effect of obesity on bone health [63].

In conclusion, the present study showed no direct correlation between BMD and vitamin D (except at lumbar spine), bone formation (PINP) and resorption (CTX1) markers, and PTH and calcium (except at hip). There were also no differences in mean values of vitamin D between control and osteoporosis postmenopausal women. The apparent protective effect of obesity on osteoporosis based on the effect of obesity on BMD masks the effect of obesity on vitamin D, PTH, and obesity-induced insulin resistance. Some of the limitations of this study were the absence of external quality control for vitamin D, PTH, and bone turnover markers and the absence of local data base to be used for identifying subjects with osteoporosis. Also, fracture data was obtained from recruited subjects not because of the absence of medical records. More focused studies are needed to understand the interaction between these factors and other signaling and genetic factors on osteoporosis.
